# Left atrial enlargement and clinical considerations in patients with or without a residual interatrial shunt after closure of the left atrial appendage with the WATCHMAN™-device

**DOI:** 10.1186/s12872-017-0728-6

**Published:** 2017-12-12

**Authors:** Blerim Luani, Thomas Groscheck, Conrad Genz, Ivan Tanev, Thomas Rauwolf, Joerg Herold, Senad Medunjanin, Alexander Schmeisser, Rüdiger C. Braun-Dullaeus

**Affiliations:** Department of Internal Medicine, Division of Cardiology and Angiology, Magdeburg University, Leipzigerstr. 44, 39120 Magdeburg, Germany

**Keywords:** LAA closure, Watchman device, Left atrial volume, 3D transthoracic echocardiography, Device related thrombi, Interatrial shunt

## Abstract

**Background:**

Interventional closure of the left atrial appendage (LAA) in patients with non-valvular atrial fibrillation, high thromboembolic and bleeding risk or bleeding history is an alternative therapeutic strategy to oral anticoagulation. It is not known if the exclusion of the LAA from the blood circulation affects the left atrial volume (LAV) and consequently its prognostic value or the circulatory performance of the heart in humans.

**Methods:**

We aimed to prospectively assess potential changes in baseline LAV, left ventricular ejection fraction (LVEF), NT-proBNP-level and the covered distance in the 6-min walk-test 6 weeks and 6 months after LAA closure with the WATCHMAN™ device. We used serial 3-dimensional transthoracic and transesophageal echocardiography to assess LAV, residual interatrial shunt and device performance in 58 consecutive patients with successful LAA closure.

**Results:**

Accurate 3D–echocardiographic data for LAV measurements were evaluable for 51 (91%) patients. Maximum LAV (LAVmax) at baseline was 102.8 ± 30.8 ml and increased significantly to 107.7 ± 32.8 ml after 6 weeks (*p* < 0.01) and 113.5 ± 34.2 ml after 6 months (*p* < 0.01). Minimal LAV (LAVmin) increased from 76.9 ± 29.5 ml at baseline to 81.8 ± 30.2 ml after 45 days (*p* < 0.01) and 82.1 ± 33.3 ml after 6 months (*p* < 0.01). Similarly, their indexes to BSA (LAVImax and LAVImin) increased significantly, as well. Patients without a residual left-to-right interatrial shunt showed a significantly higher increase in LAVmax or LAVmin. Baseline LVEF, NT-proBNP-level or the distance covered at the 6-min walk test did not significantly change 6 weeks or 6 months after LAA closure.

**Conclusions:**

LAVmax and LAVmin increase significantly after interventional LAA closure. LA enlargement does not correlate with clinical progression of heart failure. Persistent left-to-right interatrial shunt counteracts the LA enlargement. A reduced LA compliance after exclusion of the LAA from the blood circulation with consecutive increase in LA pressure may be a potential cause of LA enlargement and warrants further investigation.

**Trial registration:**

German Clinical Trials Register ID: DRKS00010768; Registration Date 07.07.2016.

## Background

The interventional closure of the left atrial appendage (LAA) in patients with non-valvular atrial fibrillation, moderate or high thromboembolic risk and a high bleeding risk or bleeding history is an alternative therapeutic strategy to oral anticoagulation [1–5]. The long-term results of a randomized study [6] and a meta-analysis [7] showed reduced cardiovascular mortality and non-inferiority regarding all-cause stroke after interventional LAA closure compared to oral anticoagulation. Assuming that the pathomechanism of the added risk for stroke in these patients is the embolism of intracardiac thrombi, the rationale behind the procedure is to close the site of the most frequent thrombus formation, since 90% of thrombi in patients with non-valvular atrial fibrillation are found in the LAA [8].

The main task of the LAA is thought to be maintenance of hemodynamic homeostasis and neurohumoral response to fluid overload by enhanced secretion of natriuretic peptides, known as “stretch-secretion coupling” [9]. This is consistent with the independent findings of Spencer and Tabata showing that the LAA distention depends on volume loading and predicts A-type natriuretic peptide (ANP) levels in humans [10, 11]. While using different imaging modalities in clinical practice large differences in LAA shape and volume are observed, sometimes finding that LAA makes up to one third of the left atrial (LA) volume. In a previous anatomical study, plastic casts of human LAA post mortem revealed variable shapes and volume-ranges [12] with a larger mean volume in patients with atrial fibrillation as compared to those in sinus rhythm. Hoit et al. showed in an animal study that the LA compliance, conduit function and the relative reservoir to conduit function decrease after surgical ligation of the LAA [13, 14]. In another study, Providencia et al. demonstrated that the enlargement and dysfunction of the LA predict cardiovascular events in patients with non-valvular atrial fibrillation [15]. Up to date, it is not known, if the exclusion of the LAA volume from the blood circulation after interventional closure using an occluder device in humans affects the volume and function of the LA and consequently the circulatory performance of the heart, the exercise-capacity of the patients as well as the prognostic significance of these parameters.

We aimed to prospectively assess potential changes in LA volumes and their clinical significance after interventional LAA closure with the WATCHMAN™ Device using serial 3-dimensional transthoracic and transesophageal echocardiography (3D TTE and TEE).

## Methods

Consecutive patients with non-valvular atrial fibrillation, moderate or high thromboembolic risk and contraindication for anticoagulation therapy or high bleeding risk, who were referred to our institution for interventional LAA closure, were enrolled in our study after giving their written informed consent. Patients underwent a diagnostic evaluation before the implantation procedure as well as 6 weeks and 6 months thereafter. All evaluations included 3D TTE and TEE, a six-minutes walk test, 24-h blood pressure monitoring, 12-lead and 24-h Holter electrocardiography (ECG), blood sampling for routine laboratory tests, drug history and the assessment of cerebrovascular events or bleeding complications.

### LAA closure

All Patients received a 3rd generation WATCHMAN™ or 4^th^ generation WATCHMAN FLX™ Left Atrial Appendage Closure (LAAC) device (Boston Scientific, Marlborough, USA). We described the device in a previous study in detail [16]. The size of the device was selected according to the recommended compression in relation to the size of the LAA. Care was taken to avoid a residual leakage of > 5 mm between the occluder device and atrial wall. All patients received a dual antiplatelet therapy, which was limited to acetylsalicylic acid monotherapy 100 mg per day after exclusion of device thrombi at the scheduled TEE control after 6 months, according to our institutional routine practice.

### Echocardiography

**Fig. 1:**
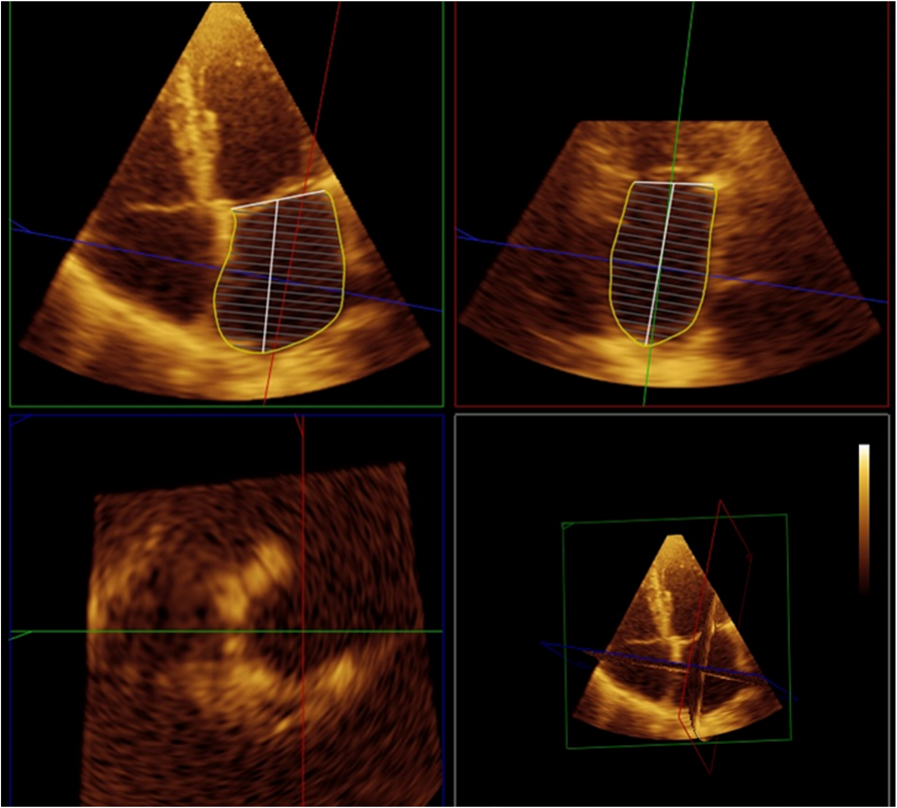
Left atrial volume measurement by 3D transthoracic echocardiography. Left atrial volumes (LAV) were measured by freehand 3D–transthoracic echocardiography using a dedicated software for left ventricular analysis (QLAB, 8.1, Philips Deutschland GmbH, Hamburg, Germany). Care was taken to manually adjust the measurement planes appropriately and exclude the pulmonary vein ostia and the left atrial appendage from the LAV at baseline, as well as at follow up controls after the procedure

We performed real-time 3D TTE using a commercial ultrasound system (iE33 xMATRIX, Philips Deutschland GmbH, Hamburg, Germany) equipped with an X3–1 probe to assess LA maximum (LAVmax) and minimal (LAVmin) volumes in all patients as well as the pre- atrial contraction volume (LAVpreA) in patients with sinus rhythm. Multibeat (6–8) sequences of the LA at standard apical views were acquired in left lateral decubitus at breath holding in end-expiration to avoid motion of the probe and were digitally recorded for later offline analysis. LA volumes were measured by freehand 3D echocardiography as described elsewhere [17] using a software dedicated for left ventricular analysis (QLAB, version 8.1, Philips Deutschland GmbH, Hamburg, Germany), Fig. 1. LAA or the ostia of the pulmonary veins were excluded from the LA volume. All measurements were performed by two investigators blinded to baseline or clinical parameters. The measurements of the echocardiographic parameters from the 6 week and 6 month follow-up were performed with a time delay of 2 weeks to the previous measurement. LAVmax was assessed in end-systole just before the opening of the mitral valve, whereas LAVmin was assessed in end-diastole at mitral valve closure. In patients with sinus rhythm LAVpreA was assessed additionally adjacent to the P-wave onset. In patients with atrial fibrillation at the time of the echocardiographic examination mean LA volumes were calculated out of five cardiac cycles.

The position and fitting of the occluder device, thrombus formation, residual leakage between LAA wall and occluder device, residual interatrial shunting after transseptal puncture and mitral valve insufficiency were assessed by 2D TEE.

### Statistics

Categorical parameters are presented as counts and percentages, continuous variables are presented as mean values ± SD. Categorical variables were compared by chi-square test, continuous variables were compared using the matched-pairs t-test after controlling for normal distribution using the Kolmogorov-Smirnov test.

Left atrial volume index (LAVI) was calculated as a quotient of left atrial volume (LAV) and body surface area (BSA). Correspondingly, LAVImax, LAVImin or LAVIpreA were calculated as a quotient of LAVmax, LAVmin and LAVpreA to BSA. BSA was calculated using the DuBois and DuBois formula.

A *p*-value <0.05 was considered statistically significant. The significance level for multiple comparisons was adjusted by Bonferroni. Statistical analysis was performed using SPSS Version 22 (IBM, Armonk, NY, USA).

## Results

Interventional LAA closure was successful in 58 (94%) of 62 consecutive patients, no procedure related major complications were observed. Two patients received two LAAC devices each, due to a special LAA anatomy with two and three prominent lobes, respectively, in which a single occluder couldn’t seal both or all three lobes. Two patients died before the 6 week follow up visit of myocardial infarction and decompensated congestive heart failure, respectively. Accurate echocardiographic data for serial LAV measurements were evaluable for 51 (91%) patients. Mean age was 74.4 ± 7.6 years, CHA_2_DS_2_VASc Score 3.2 ± 1.5 and HAS-BLED Score 3.1 ± 1.1. Clinical indication for interventional LAA closure was a previous bleeding complication in 24 (47.1%) or a high bleeding risk in 26 (51%) patients, whereas 1 (2%) patient had a profession related limitation to oral anticoagulation therapy. Baseline clinical parameters of the study population are shown in Table 1.

**Table 1:** Patient baseline characteristics

Patients available for analysis, *n*	51
Mean age (years)	74.4 ± 7.6
Male, n (*%*)	35 (68.6)
CHA_2_DS_2_-VASc score	3.2 ± 1.5
HAS-BLED score	3.1 ± 1.1
Body mass index (kg/m^2^)	28.8 ± 5.7
Atrial fibrillation persistent or permanent, n (%)	40 (78.4)
*Clinical indication for LAA closure*	
Previous bleeding complication, n (%)	24 (47.1)
• Intracranial bleeding, n (%)	3 (5.9)
• Gastrointestinal bleeding, n (%)	13 (25.5)
• Other bleeding, n (%)	8 (15.7)
High bleeding risk, n (%)	26 (51.0)
Other limitation to oral anticoagulation therapy, n (%)	1 (2.0)
*Echocardiographic characteristics*	
Interventricular septum thickness (IVS), mm	10.4 ± 1.5
Left ventricular ejection fraction (LVEF), %	49.5 ± 12.2
Mitral regurgitation, n (%)	
no or mild	38 (74.5)
moderate	13 (25.5)
severe	0
*Medication, n (%)*	
Beta-blocker	20 (39.2)
ACE inhibitor/AT-blocker	23 (45.1)
Loop diuretic	32 (62.7)

### Echocardiographic results

LAVmax at baseline was 102.8 ± 30.8 ml and increased significantly to 107.7 ± 32.8 ml after 6 weeks (*p* < 0.01) and 113.5 ± 34.2 ml after 6 months (*p* < 0.01). LAVmin increased from 76.9 ± 29.5 ml at baseline to 81.8 ± 30.2 ml after 6 weeks (*p* < 0.01) and 82.1 ± 33.3 ml after 6 months (*p* < 0.01). Similarly, LAVImax and LAVImin increased significantly after 6 weeks and 6 months (for details see Table 2). From initially 11 patients at baseline, only 8 and 5 patients were still in sinus rhythm after 6 weeks and 6 months, respectively. In these limited patient subgroups LAVpreA increased from 77.3 ± 23.6 ml at baseline to 80.2 ± 26.2 ml after 6 weeks and 80.3 ± 30.1 ml after 6 months (*p* = 0.15 and *p* = 0.053, respectively). The difference between LAVmax and LAVpreA or the ratio (LAVmax-LAVpreA)/LAVmax were unchanged at 6 week or 6 month follow up, as compared to baseline. The difference between LAVpreA and LAVmin did not change after 6 weeks or 6 months, as well.

**Table 2:** Left atrial volume measurements at six weeks and six months, compared to baseline, respectively

	Baseline	6 weeks	p^1^	6 months	p^2^
(*n* = 51, if not specified)					
LAVmax, ml	102.8 ± 30.8	107.7 ± 32.8	< 0.01	113.5 ± 34.2	< 0.01
LAVpreA, ml (6we:*n* = 8; 6mo: *n* = 5)	77.3 ± 23.6	80.2 ± 26.2	0.15	80.3 ± 30.1	0.05
LAVmin, ml	76.9 ± 29.5	81.8 ± 30.2	< 0.01	82.1 ± 33.3	< 0.01
LAVImax, ml/m^2^	53.6 ± 17.0	56.1 ± 17.8	< 0.01	59.7 ± 17.7	< 0.01
LAVIpreA, ml/m^2^. (6we:n = 8; 6mo: *n* = 5)	41.56 ± 17.2	43.1 ± 18.3	0.14	46.7 ± 21.6	0.05
LAVImin, ml/m^2^	40.2 ± 16.2	42.5 ± 16.1	< 0.01	43.3 ± 17.6	< 0.01

p^1^ = *p* value (comparison between six weeks and baseline measurements); p^2^ = *p* value (comparison between six months and baseline measurements)

**Fig. 2:**
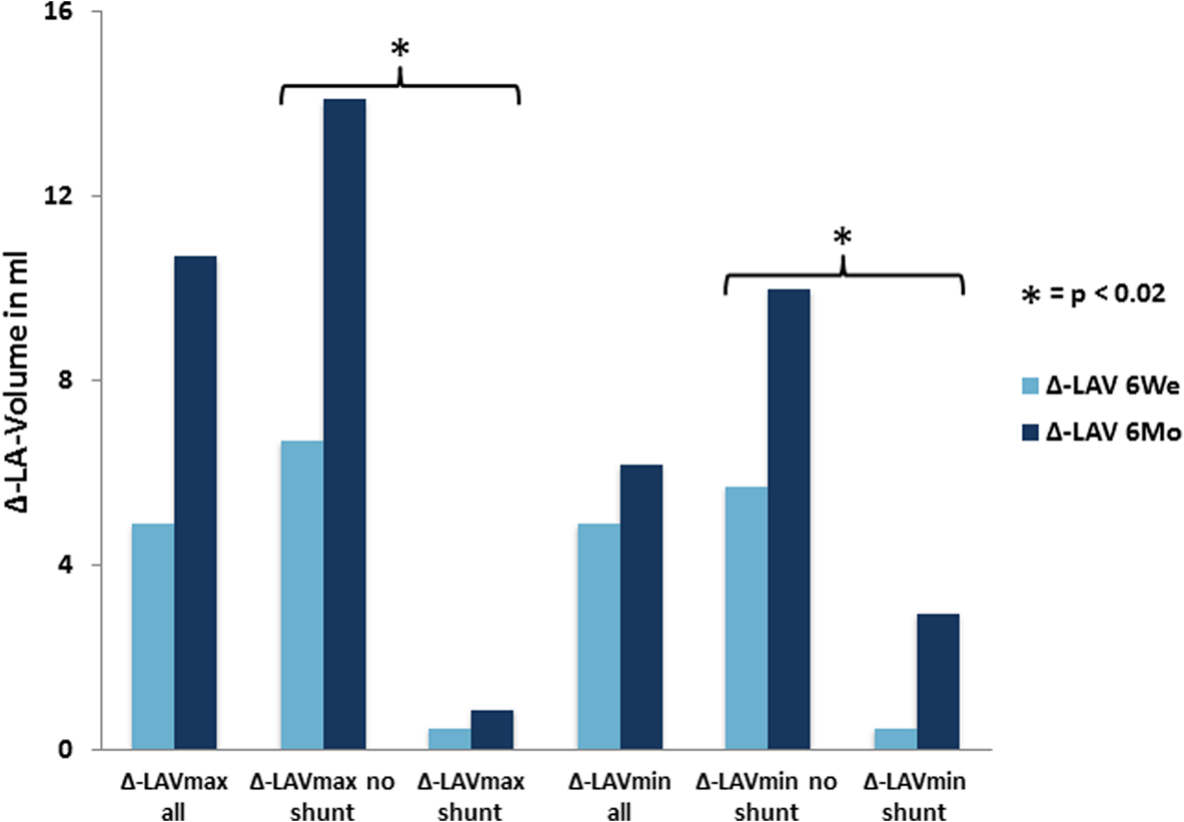
Changes in baseline left atrial volume six weeks and six months after LAA closure. Δ-LAVmax and Δ-LAVmin are shown for all patients, as well as for the subgroups with and without a residual interatrial shunt. The increase in Δ-LAVmax and Δ-LAVmin after six weeks and six months was significant in all patients. Patients with no residual atrial shunt after transseptal access showed a significantly higher increase in Δ-LAVmax or Δ-LAVmin, as compared to those with a residual one

A left-to-right interatrial shunt due to a residual septal defect after transseptal access or a large patent foramen ovale was documented in 15 and 10 patients after 6 weeks and 6 months, respectively. The increase in LAVmax after 6 weeks and 6 months was significantly higher in patients without a left-to-right interatrial shunt, as compared to those with a residual one (Δ-LAVmax after 6 weeks and 6 months in patients with no shunt: 6.72 ± 13.9 ml and 14.1 ± 17.3 ml; in those with shunt: 0.48 ± 7.4 ml and 0.89 ± 7.7 ml; *p* = 0.01 and *p* < 0.01, respectively). Δ-LAVmin after 6 weeks or 6 months was also significantly different in patients with and without interatrial shunt (Fig. 2). Δ-LAVmax or Δ-LAVmin after 6 weeks or 6 months was not dependent on LVEF or the presence of left ventricular hypertrophy, as measured by TTE at baseline.

A residual leak after successful LAA closure was seen in 16 patients after 6 weeks and 13 patients after 6 months. All residual leaks were less than 5 mm, as per quality criteria at implantation. A residual leak did not affect the LA enlargement after 6 weeks or 6 months in the study population or the subgroup without a left-to-right interatrial shunt.

LVEF did not significantly change after 6 weeks or 6 months (LVEF in %, at baseline: 49.5 ± 12.2; after 6 weeks: 49.1 ± 11.7; after 6 months: 47.9 ± 12.5; p = n.s., respectively).

A thrombus on the occluder device was primary diagnosed in 4 patients after 6 weeks and 2 more patients after 6 months, with all but one patient being on dual antiplatelet therapy at the time of diagnosis. There was a clear trend for a lower glomerular filtration rate (GFR) in patients with thrombus formation as compared to those without the latter (41.1 ± 21.9 vs. 66.0 ± 29.3 ml/min/1.73m^2^, *p* = 0.051). Patients with diagnosed thrombus on the occluder device received immediately an anticoagulation therapy with a vitamin K antagonist. Thrombus resolved without sequelae in 5 of 6 patients. The patient with a persistent thrombus on the occluder device despite anticoagulation therapy was closely monitored by serial TEE controls, which revealed constant thrombus dimensions and consistence, probably due to consolidation and endothelialization of the thrombus. No patient with a thrombus on the occluder device suffered a cerebrovascular or peripheral embolic event.

### Clinical parameters

Forty one (80.4%) patients were able to accomplish a six-minute walk test. The covered distances at baseline, after 6 weeks and 6 months were not significantly different (372 ± 96 m vs. 362.1 ± 105.8 m vs. 375.3 ± 119.0 m, respectively; p = n.s.). Mean systolic or diastolic blood pressure, potassium, sodium or GFR remained unchanged after 6 weeks or 6 months. Median NT-proBNP at baseline was 1283 pg/ml (IQR: 628–2011 pg/ml) and did not significantly change after 6 weeks (1049 pg/ml, IQR: 748 – 2271 pg/ml) or 6 months (1066 pg/ml, IQR: 610–1867 pg/ml) follow up (p = n.s., respectively).

During follow up one patient suffered an ischemic cerebrovascular event, while being on dual antiplatelet therapy, no bleeding complications occurred.

## Discussion

In our prospective trial we could demonstrate by serial real-time 3D TTE that LA maximum and minimal volume or their quotients to BSA (LAVImax and LAVImin) increase significantly after interventional LAA closure using the Watchman occluder, especially in patients without a residual interatrial shunt, which to our best knowledge has not been reported before in humans.

It has been previously reported that LA enlargement and dysfunction are important predictors of clinical outcomes such as new development of congestive heart failure or hospitalization for heart failure, cardiovascular events or all-cause mortality [18–23]. Other studies found that LAVImax is an even more sensitive predictor of clinical outcome and provides accurate risk stratification for congestive heart failure, cerebrovascular events, acute myocardial infarction and cardiovascular death [24]. However, the life expectancy of atrial fibrillation patients after interventional LAA closure is not inferior when compared to those receiving oral anticoagulation therapy. The Protect AF trial [6] showed quite the contrary with even reduced cardiovascular and all-cause mortality after interventional LAA closure compared to the medical therapy arm. A recently published meta-analysis showed that interventional LAA closure is even comparable to non-vitamin K oral anticoagulants (NOACs) and superior to placebo or antiplatelet therapy for preventing mortality [25].

The left atrium is exposed to the left ventricular filling pressure and volume or pressure overload results in LA enlargement, which is associated with complications [26, 27]. But, the increase in LA maximum and minimal volumes (as well as their indexes to BSA: LAVImax and LAVImin) in our patients do not seem to rely on a remarkable progression of conditions with heart failure, since the LVEF, NT-proBNP levels or the covered distances at the 6 min walk test did not significantly change 6 weeks or 6 months after interventional LAA closure. It is also unlikely, that the LA enlargement was mostly caused by anatomical remodeling due to atrial fibrillation, as persistent or permanent atrial fibrillation was already present in nearly 80% of patients at baseline and atrial fibrillation load was not significantly different between baseline and follow up visits.

Previously, Hoit et al. [14] demonstrated a decreased left atrial compliance in a canine model after exclusion of the LAA from the blood circulation, illustrated by a shift of the pressure-volume curve towards higher pressure and lower volumes. In a small series of 15 patients undergoing coronary artery bypass grafting or mitral valve surgery Tabata et al. reported increased transmitral flow velocities, mean LA pressure and maximal LA dimensions after LAA clamping [28], which reflect the important contribute of LAA to the left atrial reservoir function. In a recently published propensity score-matched analysis Melduni et al. reported a higher risk of early postoperative atrial fibrillation (POAF) in patients with LAA closure during routine cardiac surgery [29]; the authors postulate that the increased risk of POAF is likely mediated by LA- and pulmonary-vein-stretch, as a consequence of decreased LA compliance following LAA closure, though hemodynamic or echocardiographic measurements were not performed in this series. The findings of our study and the above mentioned data do not necessarily suggest an impaired systolic or diastolic left ventricular function or reserve as the cause of left atrial enlargement following interventional LAA closure (though it might be the key factor of LA enlargement in the general population), but rather a reduced LA compliance with consequent increase in LA pressure after LAA closure.

In a recently published series of patients with interventional LAA closure Coisne et al. reported an improved LA mechanical function, illustrated by increased LA emptying fraction and peak atrial contraction strain (derived by speckle tracking echocardiography) in sinus rhythm patients [30], probably due to a Frank-Starling effect. However, in the limited subgroups of sinus rhythm patients in our study (eight and five patients after 6 weeks and 6 months, respectively) we did not notice significant changes in the LA mechanical function.

Hasenfuß reported a significantly lower pulmonary capillary wedge pressure at rest and during exertion after transcatheter implantation of an interatrial shunt device in patients with heart failure and preserved LVEF [31]. Similarly, del Trigo showed a decrease in the pulmonary capillary wedge pressure and an improvement in the NYHA class in patients with heart failure and reduced LVEF 3 months after implantation of another left-to-right interatrial shunt device [32]. On the other hand, Ewert et al. showed that left atrial mean pressure and v-wave peak value, as a surrogate marker of atrial compliance (provided that the severity of mitral valve insufficiency does not change) increased during balloon occlusion of atrial septal defects in elderly patients with reduced diastolic elasticity [33]. In our study population fifteen and ten patients showed a spontaneous left-to-right interatrial shunt after 6 weeks and 6 months, respectively. The increase in LA volume in the subgroup of patients without a residual interatrial shunt was significantly higher as compared to those with a residual one. This finding supports the consideration that LA enlargement after LAA closure in our study population may have occurred as a consequence of impaired compliance with a consecutive increase in LA pressure, whereas a persistent left-to-right interatrial shunt may have mitigated the increase in that pressure, but this certainly warrants further investigation by a prospective hemodynamic study.

Taking into account the above mentioned findings and suggestions, our results should be considered when assessing LA volumes or function indexes for correct interpretation of their diagnostic and prognostic value in patients with interventional LAA closure.

Thrombus formation on the occluder device was diagnosed in 6 (11.8%) patients at either 6 week or 6 month TEE controls. None of these patients suffered a thromboembolic complication during follow up. Thrombus resolved in all but one patient after oral anticoagulation therapy; in one case thrombus consolidation was documented by serial TEE controls. The rate of diagnosed device-related thrombi in our study was higher than that reported in the PROTECT-AF Trial (11.8 vs. 5.7%) [34]. This difference may be explained by the temporary use of vitamin K anticoagulants in the PROTECT-AF Trial, as all our patients received solely a dual antiplatelet therapy up to 6 months after implantation. However, our findings emphasize the importance of mid- and long term TEE controls after interventional LAA closure. Adhering to our protocol (or to a similar one) for TEE controls, should allow for a safe use of dual antiplatelet therapy, as an appropriate antithrombotic regimen after interventional LAA closure using the Watchman device.

Impaired endothelialization has been reported for patients with chronic kidney disease after implantation of bare metal coronary stents [35]. The clear trend towards lower GFR in patients with a device related thrombus might be explained by a delayed or reduced endothelialization in patients with chronic kidney disease and consequently higher probability for thrombus formation on the occluder device, but this consideration warrants further investigation.

## Conclusion

Left atrial maximum and minimal volumes increase significantly after interventional LAA closure using the WATCHMAN™ device. LA enlargement did not correlate with conditions of progressive heart failure in our study population, since NT-proBNP levels, LVEF or the covered distances at the 6-min walk test did not significantly change 6 weeks or 6 months after the implantation procedure, as compared to baseline. This important finding may explain the reverse correlation between LA enlargement and prognosis in these patients, considering that all-cause mortality is even reduced after interventional LAA closure, as reported in the PROTECT-AF Trial. Persistent left-to-right interatrial shunt counteracts the LA enlargement. Further investigation is warranted to find out whether a reduced LA compliance and a consecutive increase in LA pressure is the main cause of LA enlargement after exclusion of the LAA from the blood circulation.

Mid- and long term TEE controls after interventional LAA closure using the WATCHMAN™ device are strongly recommended to rule out device related thrombus formation and allow for a safe management of these patients.

## Abbreviations

3D TTE: 3-dimensional transthoracic echocardiography
ANP: A-type natriuretic peptide
BSA: Body surface area
ECG: Electrocardiography
GFR: Glomerular filtration rate
LA: Left atrium
LAA: Left atrial appendage
LAAC: Left atrial appendage closure
LAV: Left atrial volume
LAVI: Left atrial volume index
LVEF: Left ventricular ejection fraction
NT-proBNP: N-terminal-pro-B-type natriuretic peptide
POAF: postoperative atrial fibrillation
TEE: Transesophageal echocardiography
